# *Ureaplasma diversum* and Its Membrane-Associated Lipoproteins Activate Inflammatory Genes Through the NF-κB Pathway via Toll-Like Receptor 4

**DOI:** 10.3389/fmicb.2018.01538

**Published:** 2018-07-12

**Authors:** Manoel N. Santos-Junior, Izadora S. Rezende, Clarissa L. S. Souza, Maysa S. Barbosa, Guilherme B. Campos, Laís F. Brito, Éllunny C. Queiroz, Elaine N. Barbosa, Mariana M. Teixeira, Letícia O. Da Silva, Lucas S. C. Silva, Flávia S. Nascimento, Tassyo L. Da Silva, Adam A. Martens, Adriano F. P. Siqueira, Mayra E. O. D’Avila Assumpção, Glaucia M. Machado-Santelli, Bruno L. Bastos, Ana M. S. Guimarães, Jorge Timenetsky, Lucas M. Marques

**Affiliations:** ^1^Department of Biointeraction, Multidisciplinary Institute of Health, Universidade Federal da Bahia, Vitória da Conquista, Brazil; ^2^Department of Microbiology, State University of Santa Cruz (UESC), Ilhéus, Brazil; ^3^Department of Microbiology, Institute of Biomedical Science, University of São Paulo, São Paulo, Brazil; ^4^Department of Cellular Biology and Development, Institute of Biomedical Sciences, University of São Paulo, São Paulo, Brazil; ^5^Department of Animal Reproduction, College of Veterinary Medicine, University of São Paulo, São Paulo, Brazil

**Keywords:** *Ureaplasma diversum*, immunogenicity, macrophages, blastocysts, LAMPs

## Abstract

**Objectives:** *Ureaplasma diversum* is a pathogen of cows that may cause intense inflammatory responses in the reproductive tract and interfere with bovine reproduction. The aims of this study were to evaluate the immune response of bovine blastocysts and macrophages to *U. diversum* infection and to evaluate the invasion capacity of this microorganism in bovine blastocysts.

**Methods:** Viable and heat-inactivated *U. diversum* strains ATCC 49782 and CI-GOTA and their extracted membrane lipoproteins were inoculated in macrophages in the presence or absence of signaling blockers of Toll-Like Receptor (TLR) 4, TLR2/4, and Nuclear Factor KB (NF-κB). In addition, the same viable *U. diversum* strains were used to infect bovine blastocysts. RNA was extracted from infected and lipoprotein-exposed macrophages and infected blastocysts and assayed by qPCR to evaluate the expression of Interleukin 1 beta (IL-1β), Tumor Necrosis Factor Alpha (TNF-α), TLR2 and TLR4 genes. *U. diversum* internalization in blastocysts was followed by confocal microscopy.

**Results:** Both *Ureaplasma* strains and different concentrations of extracted lipoproteins induced a higher gene expression of IL-1β, TNF-α, TLR2, and TLR4 in macrophages (*p* < 0.05) when compared to non-infected cells. The used blockers inhibited the expression of IL-1β and TNF-α in all treatments. Moreover, *U. diversum* was able to internalize within blastocysts and induce a higher gene expression of IL-1b and TNF- α when compared to non-infected blastocysts (*p* < 0.05).

**Conclusion:** The obtained results strongly suggest that *U. diversum* and its lipoproteins interact with TLR4 in a signaling pathway acting via NF-kB signaling to stimulate the inflammatory response. This is the first study to evaluate the *in vitro* immunological response of macrophages and bovine blastocysts against *U. diversum*. These results may contribute to a better understanding of the immunomodulatory activity and pathogenicity of this infectious agent.

## Introduction

*Ureaplasma diversum* has been reported to infect the respiratory and genital tracts of cattle and cause a myriad of reproductive alterations, including granular vulvovaginitis, endometritis, salpingitis, infertility, abortion, and changes in spermatozoa morphology (Marques et al., 2011; Hobson et al., 2013). Despite these reports, studies on the relationship of *U. diversum* and bovine reproductive disorders have been regarded controversial mainly due its finding in healthy animals (Taylor-Robinson et al., 1967; Howard et al., 1973; Ball and Mackie, 1985; Buzinhani et al., 2007b; Marques et al., 2011). Nevertheless, the process that leads to infertility or pregnancy failures in *U. diversum* infected cows is believed to occur due to the pathogen’s damage to the oocyte, uterus and epithelium of the oviduct, which may interfere with the embryo development and possibly lead to embryonic death (Doig et al., 1980; Britton et al., 1987; Lewandowska-Sabat et al., 2013). On the other hand, abortion in cattle by *U. diversum* may be caused by placental and fetal pneumonia mainly in the last trimester of pregnancy (Ibrahim et al., 2015).

In general, *Mollicutes* have a diversity of lipid-associated membrane proteins (LAMPs) that are strongly associated with the virulence and pathogenicity of these bacteria (Chambaud et al., 1999; You et al., 2006; Wang et al., 2016). The LAMPs can modulate apoptosis (Hopfe and Henrich, 2008), the functionality of the ABC transporters (Schmidt et al., 2007) and cell adhesion (Sachse et al., 2000; Bolland and Dybvig, 2012). The recognition of LAMPs and other membrane components of some *Ureaplasmas* by cell-associated Toll Like Receptors (TLRs) have been shown to modulate the cytokine release of immune cells (Wang et al., 2016). The signaling pattern and activated TLRs, however, may differ among different *Mollicutes* *species* (Peltier et al., 2007; Shio et al., 2014; Wang et al., 2016). In general, bacterial LAMPs interact with specific TLRs and recruit intracellularly the myeloid differentiation factor 88 (MyD88) leading to activation of NF-κB and the protein 1 (AP-1) (He et al., 2009; Wang et al., 2016). NF-κB and AP-1 regulate important genes associated with inflammation in infection processes (Garcia et al., 1998; Rawadi et al., 1999). Accordingly, the recent genome analyses of *U. diversum* revealed a large number of genes encoding membrane-associated lipoproteins, as well as urease, hemolysin, phospholipase and glycosyltransferase (associated with capsule synthesis) enzymes (Marques et al., 2015, 2016). These lipoproteins, however, have never been separately analyzed for their host immune system stimulation.

Recent studies have shown that inflammatory responses to *Ureaplasma* infection play an important role in the reproductive disorders seen in cattle (Chelmonska-Soyta et al., 1994; Cardoso et al., 2000b; Buzinhani et al., 2007b; Marques et al., 2010, 2013). In normal conditions, the role of cytokines during the pre-implantation of bovine embryo and the post-fertilization process is to change the nutritional environment of the blastocyst. Thus, changes in cytokine release that can be caused by bacterial infection are likely to result in reproductive failures. As *U. diversum* has been shown to modulate cytokine release and apoptosis of murine macrophages and Hep-2 cells and to infect the *Zona pellucida* of oocysts (Britton et al., 1987; Marques et al., 2010; Buzinhani et al., 2011), it is possible that its presence in the female genital tract may alter cytokine levels and disturb the nutritional environment of the blastocyst. The immunomodulatory effects of *U. diversum* infection in blastocysts, however, have never been evaluated. In addition, *U. diversum* has been recently shown to invade Hep-2 cells and bovine spermatozoids *in vitro* (Marques et al., 2010, 2011; Hobson et al., 2013). It is likely that this intracellular activity hampers antibiotic action and the host immune response against *U. diversum* in infected animals (Amorim et al., 2014). No study to date has evaluated the invasion capacity of *U. diversum* in bovine cells of the female reproductive tract. Therefore, the aims of this study were to evaluate the immune response of bovine macrophages and blastocysts at *U. diversum* exposure and to evaluate the invasion capacity of this microorganism in bovine blastocysts.

## Methods

### *U. diversum* Culture and Inactivation

*Ureaplasma diversum* ATCC 49782, a strain from a cow with granular vulvovaginitis isolated in Ontario, Canada (Ruhnke et al., 1978), and *U. diversum* strain CI-GOTA, an isolate recovered from the vulvo-vaginal mucus of a cow in São Paulo, Brazil were used in this study. *Ureaplasmas* were cultured initially in 1 ml and expanded to 200 mL of *Ureaplasma* medium (UB) (Ruhnke et al., 1978) at 37°C. DNA was extracted from expanded cultures to confirm the presence of *U. diversum* with a species-specific PCR methodology using primers UD3 and UD4 (Cardoso et al., 2000a). At the logarithmic growth (colorimetric broth changes), the culture was centrifuged at 21,400 × *g* for 25 min at 20°C. The pellet was homogenized in phosphate buffered saline (PBS) (Silva et al., 2016) and the culture suspension was quantified in 96 well microplates to obtain an inoculum based on Colorimetric Change Units (*Ureaplasma*/mL) (Kim et al., 1994). 10^6^, 10^5^, and 10^4^ *Ureaplasma*/mL for *U. diversum* ATCC 49782 and 10^5^, 10^4^, and 10^3^ *Ureaplasma*/mL for *U. diversum* CI-GOTA were used in this study. Another fraction of the expanded culture (referring to the highest *Ureaplasma*/mL of each strain) was inactivated at 100°C for 30 min.

### Extraction of *U. diversum* Lipid-Associated Membrane Proteins (UdLAMPs)

UdLAMPs extraction followed the methodology described by He et al (He et al., 2009). Briefly, 200 mL of *Ureaplasma* culture was concentrated at 22,700 × *g* for 30 min at 4°C. The pellet was washed twice with PBS for 15 min at 4°C. The pellet was homogenized in 5 mL of Tris-EDTA followed by the addition of Triton TX-114 to a final concentration of 2%. The suspension was incubated at 48°C for 60 min. The lysate was incubated at 37°C for 10 min for phase separation. The upper aqueous phase was removed and the organic phase was completed to 1 mL by addition of Tris-EDTA, followed by addition of 2.5 mL of ethanol (ice-cold) and incubated overnight at −20°C for precipitation of the UdLAMPs. The precipitated material was centrifuged at 22,700 × *g* for 15 min at 4°C and excess of ethanol was evaporated. UdLAMPs were homogenized in PBS and quantified by the fluorimetric assay on Qubit^®^ 2.0 equipment (Thermo-Fisher, Brazil), according to the manufacturer’s instructions. The obtained UdLAMPs were visualized in acrylamide gel electrophoresis with sodium dodecyl sulfate (SDS-PAGE) and the gel was subsequently stained by Coomassie brilliant blue (Laemmli, 1970).

### Bovine Macrophage Assays

#### Isolation of Bovine Macrophages

Bovine peripheral blood was collected from the jugular vein in EDTA vacutainer and diluted 1:1 in PBS (pH 7.4) (Gondaira et al., 2015). A total of 10 mL were added in a tube over 3 ml Ficoll-Histopaque (density: 10771 g/mL, Sigma-Aldrich, Brazil) to produce the Ficoll-Histopaque barrier. This suspension was centrifuged at 4,200 × *g* at room temperature for 20 min and the mononuclear cells present at the Ficoll/plasma interface were removed and washed in PBS twice at 4,200 × *g* for 10 min. For monocyte isolation, mononuclear cells were added in solution A (5 mL of RPMI 1640 medium containing 10% fetal bovine serum) and was slowly mixed to 5 mL of solution B (5 mL of RPMI 1640 medium + 4.75 mL of Percoll + 0.322 mL of PBS). This suspension was centrifuged for 30 min (4,200 × *g*, 20°C). The monocytes were removed between the interface of both solutions (Cruz, 2010), counted in Neubauer chamber and the cell viability was assessed with 0.1% Trypan Blue (viability > 90%). Viable cell concentration was adjusted to 4 × 10^5^ cells/mL by addition of RPMI 1640 (Gibco, Brazil) medium (with 10% fetal bovine serum – Gibco, Brazil) and cultured in the same medium. After 6 days, the cell cultures were washed and after 24 h of isolation, the cells were used. The differentiation of monocytes to adherent macrophages was confirmed by microscopy (Almeida and Rosenbusch, 1991; Gondaira et al., 2015).

#### Inoculation of Bovine Macrophages With *Ureaplasma* and Extracted-Lipoproteins

Bovine macrophages (4 × 10^5^ cells/mL) were inoculated with live *U. diversum* strains ATCC49782 (10^6^, 10^5^, and 10^4^ *Ureaplasma*/mL) and CI-GOTA (10^5^, 10^4^, and 10^3^ *Ureaplasma*/mL), as well with inactivated *U. diversum* strains ATCC49782 (inactivated from 10^6^ *Ureaplasma*/ml) and CI-GOTA (inactivated from 10^5^ *Ureaplasma*/mL). Infected monolayers were incubated for 6, 12, and 24 h at 37°C in 5% CO_2_. Regarding the extracted-lipoproteins, macrophages were inoculated with UdLAMPs from both strains at concentrations of 2.0, 1.5, 1.0, and 0.5 μg/mL for 2, 6, and 12 h. Macrophages inoculated with PBS and 100 ng/mL of lipopolysaccharides (LPS - Lipopolysaccharides from *Escherichia coli* O111:B4, Sigma-Aldrich, Brazil) served as negative and positive controls, respectively. At each time, cells were collected, suspended in RNAlater^TM^ (Invitrogen, Brazil) and frozen at −70°C for subsequent mRNA extraction. All experiments were performed in triplicate with three independent repetitions (*n* = 9).

#### Signaling Inhibitors

Bovine macrophages were incubated with both viable *Ureaplasmas* (10^5^ *Ureaplasma*/mL) and UdLAMPs at 2.0 μg/ml in the presence or absence of signaling inhibitors [TLR-2/4 (OxPAPC, 10 μg/mL, Invivogen), TLR-4 (CLI-095, 1 μg/mL, Invivogen) and NF-κB (Celastrol, 5 μM, Invivogen)] using the same procedures described above (Majumder et al., 2014). The cells were analyzed after 2 h (UdLAMPs) and after 12 h (viable *Ureaplasmas*). These inhibitors were purchased from Invivogen (Invivogen, San Diego, CA) and used according to the manufacturer’s instructions. For negative controls, the cells were incubated with PBS. For positive controls, were used the synthetic bacterial lipopeptide *N*-palmitoyl-*S*-(2,3-bis(palmitoyloxy)-(2R,S)-propyl)-(R)-cysteinyl-seryl-(lysyl)3-lysine (Pam3CysSK4, EMC Microcollections GmbH, Tübingen, Germany), a selective TLR2 agonist, and LPS, a TLR4 agonist. All experiments were performed in triplicate with three independent repetitions (*n* = 9).

### Bovine Blastocyst Assays

#### Production of Embryos

Bovine ovaries were obtained from a commercial slaughterhouse and washed several times with sterile saline (0.9% [w/v] NaCl containing 100 U/ml penicillin-G and 100 mg/ml streptomycin) at 25–30°C. Cells were collected by aspirating visible follicles, and allowed to settle for 5 min. COCs (cumulus oophorus complex) were selected by their morphological appearance, including the cytoplasm aspect, and number of granulosa cell layers. Groups of 20–30 COCs were matured in 90-μl droplets of supplemented TCM-199 hepes, 0.5 μg/ml follicle-stimulating hormone - FSH (Folltropin-V; Bioniche, Belleville, Canada), 100 IU/ml human chorionic gonadotropin (Chorulon, Merck Animal Health, Boxmeer, Netherlands), and 1.0 μg/ml estradiol (Sigma-Aldrich, Brazil) under mineral oil for 24 h at 38.5°C and 5% CO_2_ with high humidity. Briefly, all *in vitro* fertilizations were performed at 5% CO_2_ in humidified air. Groups of 25 oocytes were inseminated with 1 × 10^6^/ml Percoll-purified spermatozoa from only one bull from a Brazilian Artificial Insemination Center. At approximately 20 h post-insemination, remaining granulosa cells were removed by successive pipetting. The putative zygotes were cultured in 60 μL droplets of cultive medium (KSOMaa) under mineral oil. After 3 days the medium was feeding by adding fetal bovine serum to a final concentration of 5%. The groups with 15–25 PZ were incubated until day 8 post-fertilization in saturated humidity at 38.5°C and atmosphere with 5% CO_2_, 5% O_2_, and 90% N_2_. Cleavage rates were assessed after 48 hpc and blastocyst rates were recorded after 168 hpc. After 8 days of culture in KSOM medium, blastocysts were washed in 3 drops of gentamicin-free SOF medium to remove antibiotic residues from the medium. They were classified according to the experimental group (control, ATCC 49782 and CI-GOTA) and were placed in plates with 60 μL drop of gentamycin-free SOF medium (hatched blastocyst, expanded blastocyst, blastocyst, early blastocyst) and incubated at 38.5°C in a humidified atmosphere of 5% CO_2_.

#### Blastocysts Infection

In each 60 μL drop of gentamicin-free SOF medium with 5–18 blastocysts (5 blastocysts for confocal microscopy and 18 blastocysts for gene expression), the *Ureaplasma* (MOI 1:10) were inoculated. Control group was inoculated with PBS. All blastocysts were incubated under the same conditions for 24 h. An aliquot of 25 μL of the blastocyst or *Ureaplasma* broth was stored for the control of cytokine production. Then, 30 μL of SOF with gentamicin diluted to 1:100 were added and incubated for 3 h to eliminate *Ureaplasma* inside the blastocysts to compare with those not invaded the embryo. After the incubation with gentamicin, the blastocysts were removed and washed sequentially in 3 drops of 200 μL SOF with gentamicin for 5 min. All experiments were performed in triplicate with three independent repetitions (*n* = 9).

#### Confocal Microscopy

The non-infected and infected blastocysts were fixed in 4% paraformaldehyde for 60 min and stored in PBS-PVP (polyvinylpyrrolidone) at 8°C. Treatment of blastocysts with Triton X-100 0.5% for 20 min allowed cell permeabilization. After washing cells three times with PBS containing normal horse serum, cell preparations were incubated for 40 min with 0.1 ml antibody anti-*U. diversum* (polyclonal antibody produced in rabbit from Mycoplasma Laboratory Collection) at a 1:1000 dilution in PBS with 10% normal horse serum. Coverslips were washed with PBS and blocked with 1% normal horse serum (Gibco) for 20 min. Then, cells were washed with PBS and incubated for 40 min with 0.1 ml goat anti-rabbit IgG- carbocyanine dye (Vybrant Dil - Molecular Probes) at 1:2000 dilution in PBS with 10% normal horse serum (Gibco). Each incubation step was performed with gentle shaking at room temperature. Then the incubation of this system with phalloidin associated with fluorescein isothiocyanate (FITC - Molecular Probes) for 90 min labeled the actin microfilaments in green. Finally, after washing with PBS, Vecta-Shield (Vector Laboratories Inc.,) and TOPRO-3 (Molecular Probes) fluid was added to differentiate DNA nuclei of blastocysts labeling them in blue. The cells were transferred to slides covered by cover slip preventing the cell deformity. The system was sealed to avoid drying and the analysis was carried out in a Carl Zeiss LSM 10^®^ confocal microscope, equipped with Argon laser (emission 488 nm) and helium/neon (emission 543 nm).

### Gene Expression

RNA extraction was performed according to the PicoPure kit (Applied Biosystems, Brazil) instructions, with DNAse treatment, and RNA elution in 11 μL of kit eluting solution. The cDNA was obtained using a reverse-transcription (RT) from the mRNA using the SuperScript^®^ III Reverse Transcriptase kit (Applied Biosystems, Brazil). The cDNA was used in a customized SYBR Green qPCR reaction to determine gene expression of IL-1β, TNF-α, TLR2 and TLR4 genes (Qiagen-SABioscience, Brazil). The reaction was carried out using the StepOnePlus Real-Time PCR System (Applied Biosystems, Brazil). Cycling conditions were as follows: 50°C for 10 min; 95°C for 10 min; and 45 cycles of denaturation at 95°C for 15 s, annealing at 60°C for 1 min. The melting curve was evaluated at the end of the reaction to determine the specificity of the amplification. Analysis of gene expression data was performed using the 2^−ΔΔ*C*_T_^ method (Rao et al., 2013). GAPDH was used as an endogenous gene to evaluate the overall cDNA content.

### Statistical Analysis

Comparisons were performed using the non-parametric test Kruskal-Wallis followed by the Dunn post-test. All analyses were performed with GraphPad-Prism software 6.0 (GraphPad software, San Diego, CA, United States). Statistically significant differences were found with *p* values equal to or less than 0.05, using a 95% confidence interval.

## Results

### *U. diversum* Induces the Expression of IL-1β, TNF-α, TLR2 and TLR4 in Bovine Macrophages

**FIGURE 1:**
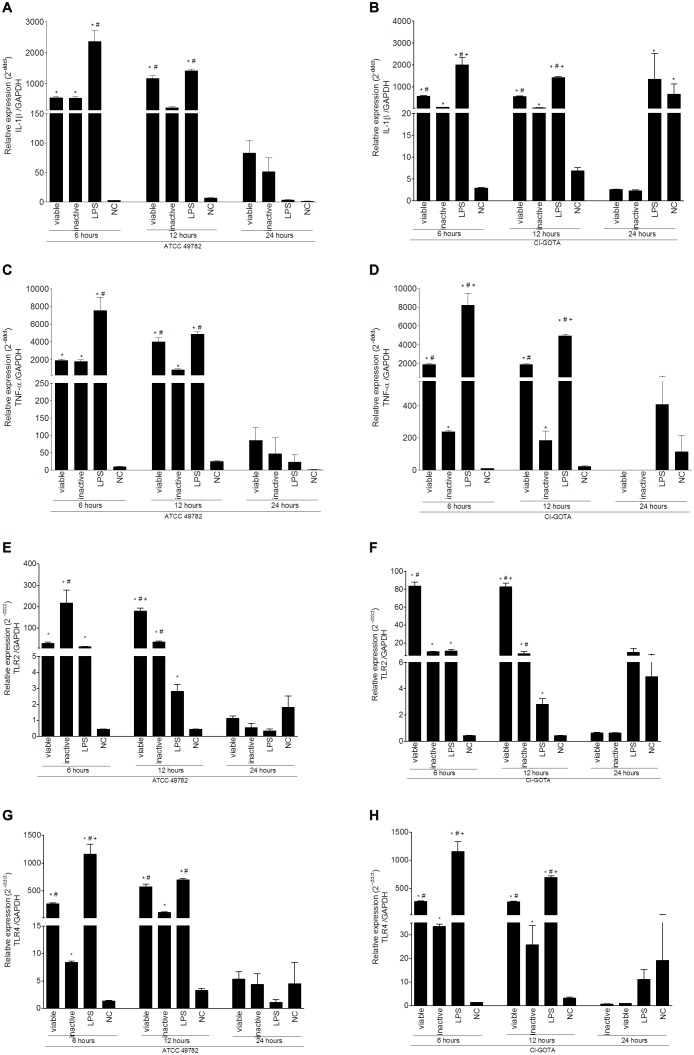
Gene expression in bovine macrophages infected with viable and inactive *U. diversum* (ATCC 49782 – 10^6^ *Ureaplasma*/mL and CI-GOTA – 10^5^ *Ureaplasma*/mL) for 6, 12, and 24 h. **(A)** IL-1β Expression induced by ATCC 49782. **(B)** IL-1β expression induced by CI-GOTA. **(C)** TNF-α expression induced by ATCC 49782. **(D)** TNF-α expression induced by CI-GOTA. **(E)** TLR2 expression Induced by ATCC 49782. **(F)** TLR2 expression in macrophages inoculated with the CI-GOTA. **(G)** TLR4 expression induced by ATCC 49782. **(H)** TLR4 expression induced by CI-GOTA. Groups were compared using the Kruskal–Wallis non-parametric test followed by the Dunn post-test. PBS was used as negative control (CN) and 100 ng/mL of LPS was used as positive control. Statistical significance (*p* < 0.05) is represented by the symbols (^∗^difference with the NC group, ^#^difference with the LPS group, ^+^difference with the inactive group). Data are expressed as mean ± standard deviation (*n* = 9).

Results from macrophage gene expression following *U. diversum* infection are shown in **Figure 1**. Loads of 10^6^ *Ureaplasma*/ml of viable (live) *U. diversum* strains ATCC49782 and 10^5^ *Ureaplasma*/ml of viable (live) CI-GOTA, as well as 10^6^ *Ureaplasma*/ml of inactivated *U. diversum* strain ATCC49782 and 10^5^ *Ureaplasma*/mL of strain CI-GOTA were used. An increased expression of IL-1β (**Figures 1A,B**), TNF-α (**Figures 1C,D**), TLR2 (**Figures 1E,F**) and TLR4 (**Figures 1G,H**) genes was observed in macrophages inoculated with viable or inactivated *Ureaplasma* (*p* < 0.05) when compared to the control (PBS) 6 and 12 h following inoculation. There were also statistical differences in gene expression of these genes in certain time-points, mainly at 6 h following infection, between viable and inactivated *Ureaplasmas* (*p* < 0.05). At 24 h following inoculation, a statistical difference between negative/positive controls and CI-GOTA strain was observed only for the IL-1β gene expression.

**FIGURE 2:**
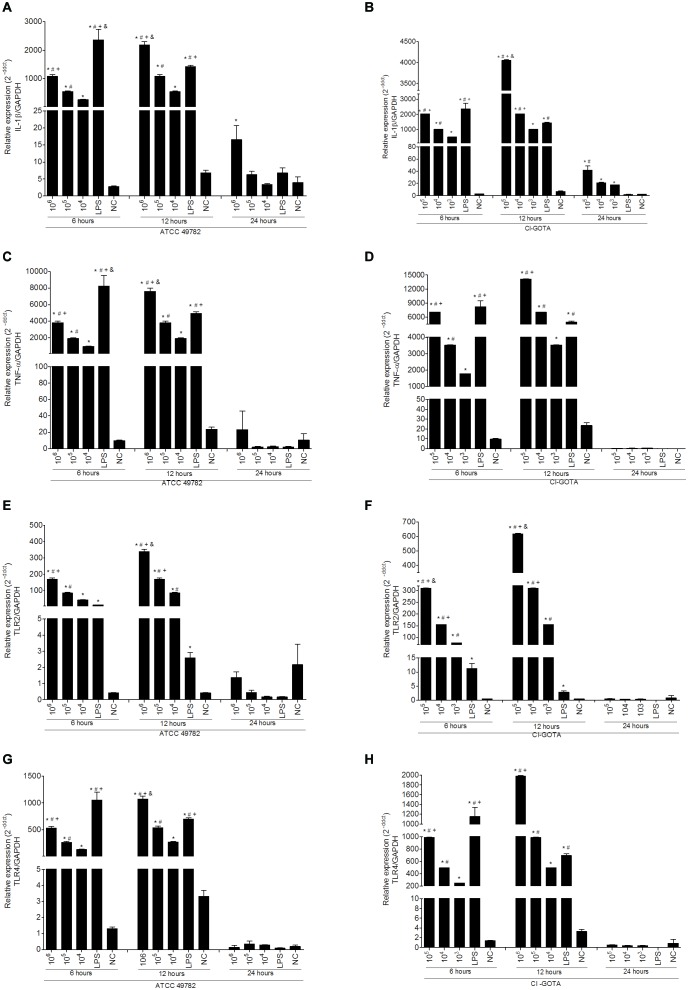
Gene expression in bovine macrophages infected with different *Ureaplasma*/mL of ATCC 49782 and CI-GOTA strains for 6, 12, and 24 h. **(A)** IL-1β expression in macrophages inoculated with the ATCC 49782 strain. **(B)** IL-1β expression in macrophages inoculated with the CI-GOTA strain. **(C)** TNF-α expression in macrophages inoculated with ATCC 49782 strains. **(D)** TNF-α expression in macrophages inoculated with CI-GOTA strain. **(E)** TLR2 expression in macrophages inoculated with ATCC 49782 strains. **(F)** TLR2 expression in macrophages inoculated with the CI-GOTA strain. **(G)** TLR4 expression in macrophages inoculated with ATCC 49782 strains. **(H)** TLR4 expression in Macrophages inoculated with CI-GOTA strain. Treatments were compared using the Kruskal–Wallis non-parametric test followed by the Dunn post-test. PBS - negative control (CN) and 100 ng/mL of LPS was used as positive control. Statistical significance (*p* < 0.05) is represented by the symbols Statistical significance (*p* < 0.05) is represented by the symbols [^∗^difference with the NC group, ^#^difference with the LPS group, ^+^difference with the 10^5^ group (ATTC) or 10^4^ group (CI-GOTA), ^&^difference with the 10^4^ group (ATTC) or 10^3^ group (CI-GOTA)]. Data are expressed as mean ± standard deviation (*n* = 9).

Results from macrophage gene expression following infection with different loads of *U. diversum* are shown in **Figure 2**. Activation of gene expression was dose dependent, i.e., the highest bacterial loads induced higher expression of the studied genes. Loads of 10^6^, 10^5^ *Ureaplasma*/ml of strain ATCC49782 and 10^5^, 10^4^, 10^3^ *Ureaplasma*/ml of strain CI-GOTA induced a statistically significant increase (*p* < 0.05) of IL-1β (**Figures 2A,B**), TNF-α (**Figures 2C,D**), TLR2 (**Figures 2E,F**) and TLR4 (**Figures 2G,H**) compared to the negative control 6 and 12 h following inoculation. A significant statistical difference (*p* < 0.05) was also observed between the different *Ureaplasma* loads of both strains and for all genes studied in these time-points. At 24 h, a statistical difference was detected only for IL-1β expression induced by the strain ATCC 49782 with 10^6^ *Ureaplasma*/mL (**Figure 2A**) and for CI-GOTA in 10^5^, 10^4^, and 10^3^ *Ureaplasma*/mL (**Figure 2B**) compared to the negative control (*p* < 0.05). Interestingly, at the same time-point of 24 h, the LPS did not induce significant genetic expression when compared to the negative control or *Ureaplasmas*.

### UdLAMPs Induce Expression of Genes Related to Pro-inflammatory Response

**FIGURE 3:**
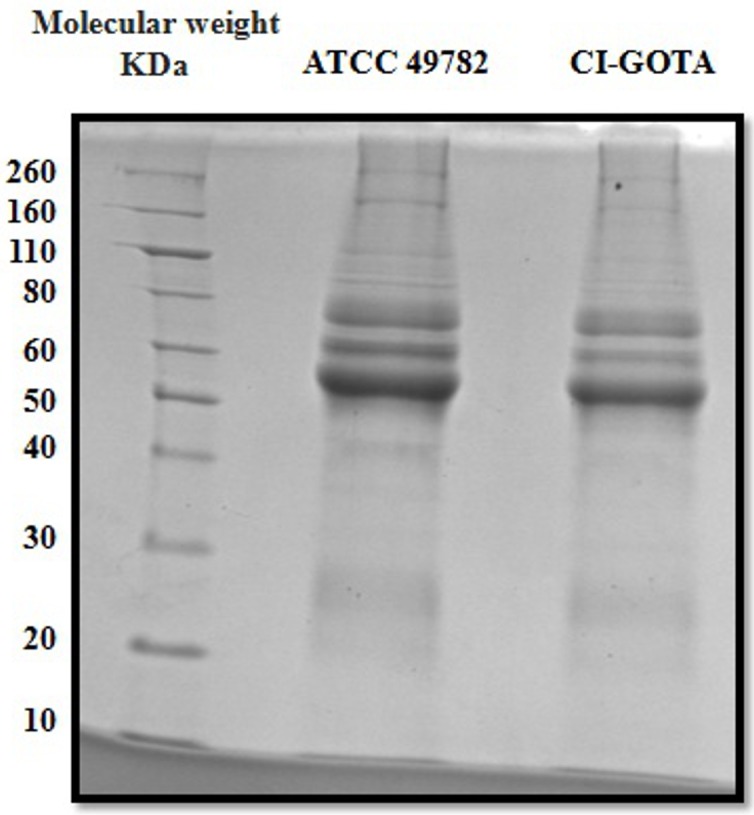
SDS-PAGE of the UdLAMPs. First column: Novex Protein molecular weight. Second column: UdLAMPs from ATCC 49782. Third column: UdLAMPs from CI-GOTA.

**FIGURE 4:**
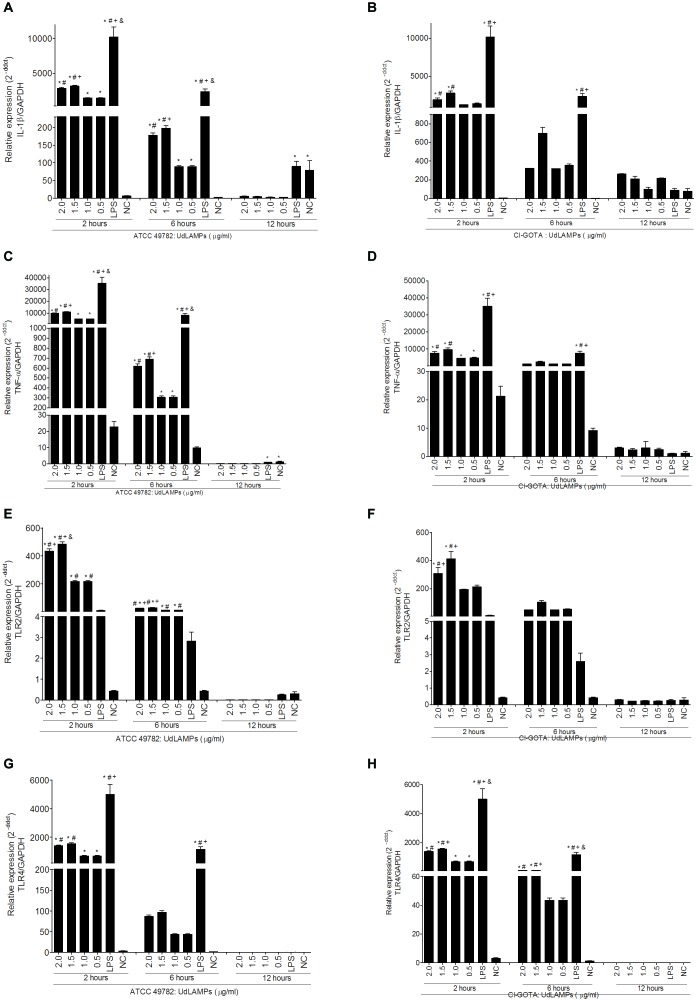
Gene expression in bovine macrophages induced by different concentrations of UdLAMPs after 2, 6, and 12 h of inoculation. **(A)** IL-1β gene expression incubation with UdLAMPs of ATCC 49782 strain. **(B)** IL-1β gene expression after incubation with UdLAMPs of the CI-GOTA strain. **(C)** TNF-α gene expression after incubation with UdLAMPs of ATCC 49782 strain. **(D)** TNF-α gene expression after incubation and others with UdLAMPs of CI-GOTA strain. **(E)** TLR2 gene expression after incubation with UdLAMPs of ATCC 49782 strain. **(F)** TLR2 gene expression after incubation with UdLAMPs of CI-GOTA strain. **(G)** TLR4 gene expression after incubation with UdLAMPs of ATCC 49782 strain. **(H)** TLR4 gene expression after incubation with UdLAMPs of CI-GOTA strain. Treatments were compared using Kruskal-Wallis non-parametric test followed by the Dunn post-test. PBS was used as negative control (CN) and 100 ng/mL of LPS was used as positive control. Statistically different groups are those presenting different symbols. Statistical significance (*p* < 0.05) is represented by the symbols (^∗^difference with the NC group, ^#^difference with the 1,5 mg/ml group, ^+^difference with the 1,0 mg/ml group, ^&^difference with the 0,5 mg/ml group). Data are expressed as mean ± standard deviation (*n* = 9).

UdLAMPs were mostly evident in sizes ranging from 50 to 80 kDa for both *Ureaplasma* strains (**Figure 3**). The band pattern observed in the SDS-PAGE corresponds to the CDSs linked to lipoproteins found in the genome of *U. diversum* ATCC49782 (Size determined using ExPASy - ProtParam tool software). UdLAMPs of *Ureaplasma* ATCC 49782 and CI-GOTA also induced higher inflammatory gene expression (*p* < 0.05) in all tested concentrations (2.0, 1.5, 1.0, and 0.5 ug/mL) (**Figures 4A–H**) when compared to the negative control. In general, the peak of expression at all concentrations tested occurred 2 h following inoculation in both strains. The exposure concentration of 1.5 ug/mL induced the highest gene expression in all cases (*p* < 0.05) when compared to the other concentrations. As observed in the analyses of the viable and inactivated conditions (**Figure 1**) and different *Ureaplasma* loads (**Figure 2**) in the 24-h period, gene expression was not significantly higher when compared to the negative controls (data not showed).

### Blockade of TLR4, TLR2 or NF-κB Inhibit the Expression of IL-1β and TNF-α in Bovine Macrophage

**FIGURE 5:**
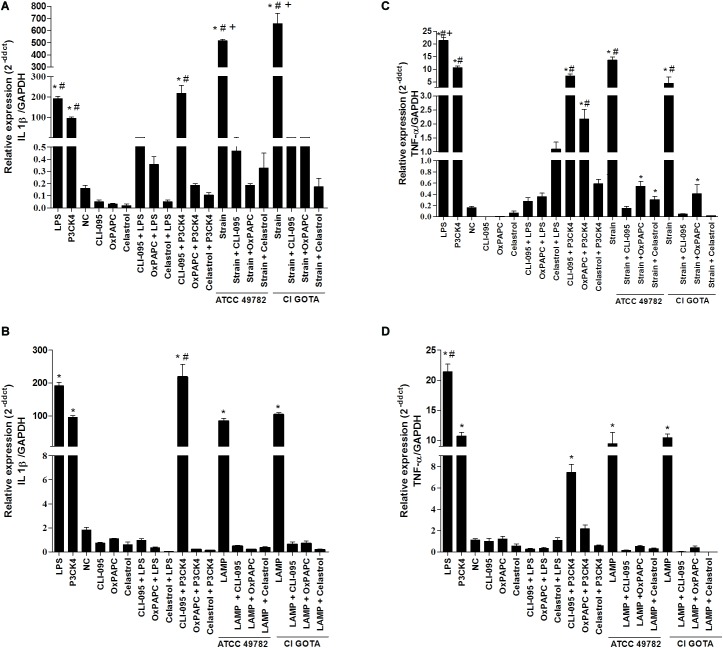
Gene expression of IL-1β and TNF-α from bovine macrophages exposed to *U. diversum* and LAMPsUd and pretreated with signaling blockers for TLR4 (CLI-095), TLR2/4 (OxPAPC), and NF-κB (Celastrol). The cells were analyzed after 2 h (LAMPs) and after 12 h (*U. diversum*). **(A)** Expression of IL-1β after incubation with ATCC 49782 (10^5^ *Ureaplasma*/mL) and CI-GOTA (10^3^ *Ureaplasma*/mL). **(B)** Expression of IL-1β after incubation with LAMPsUd of ATCC 49782 and CI-GOTA (2 μg/ml). **(C)** Expression of TNF-α after incubation with ATCC 49782 (10^5^ *Ureaplasma*/mL) and CI-GOTA (10^3^ *Ureaplasma*/mL). **(D)** Expression of TNF-α after LAMPsUd incubation of ATCC 49782 and CI-GOTA (2 μg/ml). The treatments were compared with positive control (inoculation of *U. diversum* or LAMPsUd in the absence of blocker: W/B). PBS was used as negative control (CN) and 100 ng/mL of LPS and 100 ug of Pam3CysSK4 was used as positive control. Treatments were compared using the Kruskal–Wallis non-parametric test followed by the Dunn post-test. Statistically different groups are those presenting different symbols. Statistical significance (*p* < 0.05) is represented by the symbols (^∗^difference with groups with blockers, ^#^difference with the NC group, and ^+^difference among strains). Data are expressed as mean ± standard deviation (*n* = 9).

The strains studied (ATCC 49782 and CI-GOTA) and UdLAMPs as well as TLR4 (LPS) and TLR2 (Pam3CysSK4) ligands induced greater expression of IL-1β and TNF-α when compared to the negative control and pretreated cultures with TLR4 (CLI-095), TLR2/ 4 (OxPAPC) or NF-κB (Celastrol) signaling blockers (*p* < 0.5, **Figure 5**).

### *U. diversum* Has the Ability to Invade Bovine Blastocysts

**FIGURE 6:**
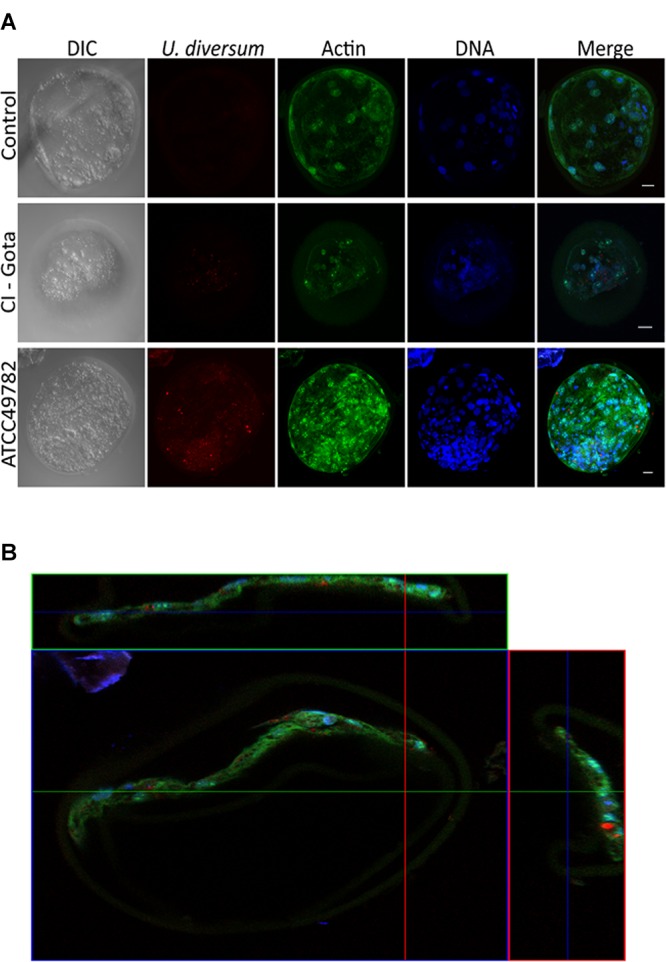
**(A)** Confocal microscopy: internalization of *U. diversum* in bovine blastocysts after 24 h of infection. Scale bars: 20 μm. *Ureaplasmas* in red (Vybrant Dil), actin in green (FITC) and DNA in blue (TOPRO-3). *U. diversum* was internalized as shown in the merged image. **(B)** Orthogonal projection of confocal microscopy showing the internalization of *U. diversum* ATCC49782 in bovine blastocysts after 24 h of infection. *Ureaplasmas* visualized by Vybrant Dil (red), actin by FITC (green) and DNA by TOPRO-3 (blue). *U. diversum* was internalized as shown in the merged image.

The exposed blastocysts (hatched and intact) to *Ureaplasma* showed internalization of the studied strains (**Figures 6A,B**). The labeled *Ureaplasmas* were in red, distinguishing them from the actin filaments and the nuclei in green and blue, respectively. Infected cells did not present cytopathic effect during the whole study. The fluorochrome Vybrant Dil also did not cause bacterial cytotoxicity, as *Ureaplasma* viability was checked after labeling. No intracellular, non-specific fluorescence of Vybrant Dil was observed in uninfected and FITC-labeled blastocysts. Images obtained in confocal microscopy varied from the basal to the apical regions of the cell.

### *U. diversum* Induces IL-1β and TNF-α Expression in Bovine Blastocysts

**FIGURE 7:**
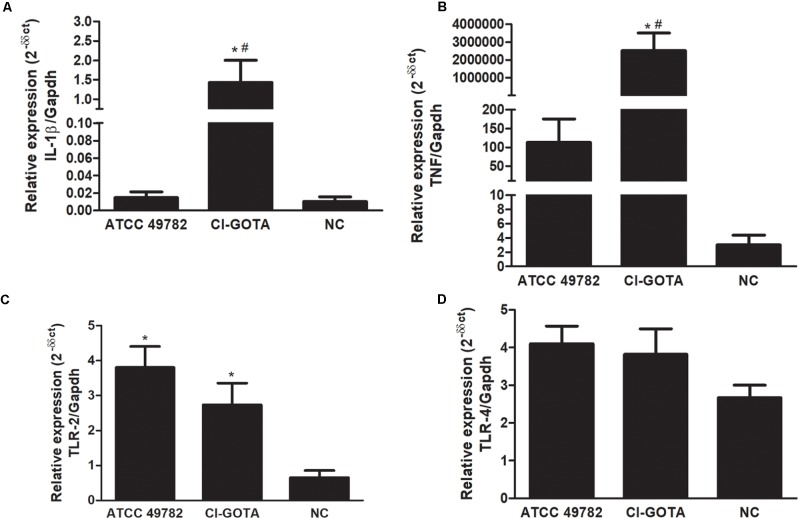
Gene expression of cytokines and TLRs in bovine blastocysts infected for 24 h with ATCC 49782 and CI-GOTA strains. **(A)** Cytokine IL-1β. **(B)** Cytokine TNF-α. **(C)** TLR 2. **(D)** TLR 4. PBS was used as negative control (CN). Statistically different groups are those presenting different symbols. Statistical significance (*p* < 0.05) is represented by the symbols (^∗^difference with the NC group, ^#^difference among strains). Data are expressed as mean ± standard deviation (*n* = 9).

Gene expression of IL-1β and TNF-α cytokines was significantly higher in the presence of the CI-GOTA strain when compared to the negative control and the ATCC49782 strain (*p* < 0.05). TLR2 gene expression was higher in blastocysts in the presence of both strains, compared with the negative control (*p* < 0.05). The same was not observed for TLR4 expression, which displayed no alteration in the gene expression in the presence of *U. diversum* (**Figure 7**).

## Discussion

The pathogenicity of *U. diversum* is not adequately understood but the activation of macrophages through TLRs has been frequently mentioned as an important feature in mycoplasmosis (Shimizu et al., 2008a; Majumder et al., 2014; Wang et al., 2016). *U. diversum* is also associated with reproductive disorders in bovine, as it causes severe inflammation, cell invasion and modulates apoptosis (Cardoso et al., 2000b; Buzinhani et al., 2007b; Marques et al., 2007, 2009, 2010, 2011; Hobson et al., 2013; Amorim et al., 2014). In the present study, inactivated and, especially, viable *U. diversum* (strains ATCC 49782 and CI-GOTA) organisms Up-Regulated gene expression of IL-1β, TNF-α, TLR2, and TLR4 in bovine macrophages. The expression of this proinflammatory gene profile induced by *U. diversum* in macrophages is supportive evidence of the severe inflammation found in the reproductive tract of infected cows (Cardoso et al., 2000a,b; Buzinhani et al., 2007a; Marques et al., 2009, 2011; Maunsell and Donovan, 2009). Murine macrophages inoculated with viable or inactivated *U. diversum* have also shown increased TLR2 gene expression and production of IL-1β and TNF-α (Marques et al., 2016). Viable or inactivated *Mycoplasma bovis* was also able to stimulate the expression of IL-1β in bovine embryo and lung cells *in vitro* (Wang et al., 2016). Further studies have shown an increase of IL-1β and TNF-α from human mononuclear cells infected with *Mycoplasma pneumoniae*, *Mycoplasma hyorhinis*, *Mycoplasma arginini*, *Mycoplasma salivarium*, *Mycoplasma orale*, and *Mycoplasma* *gallisepticum* (Cardoso et al., 2000a; Shimizu et al., 2005; Majumder et al., 2014). Thus, as observed in multiple other mycoplasma organisms, *U. diversum* induces a Pro-inflammatory state in macrophages that may be linked to disease manifestations in cattle.

In the present study, the expression of Pro-inflammatory genes following *Ureaplasma* infection was dose dependent. Silva et al. (2016) detected a higher TNF-α production in intrauterine infection with 10^6^ and 10^8^ *U. diversum*/mL and a lower response happened with 10^4^ organisms/mL (Silva et al., 2016). Likewise, *U. diversum* was also able to induce TNF-α production in mouse macrophages in a dose dependent model (Chelmonska-Soyta et al., 1994). Thus, as suggested by Marques et al. (2013), the bacterial load of *U. diversum* could be directly related to more severe clinical conditions in infected cows, which may have implications in disease transmission and control.

The UdLAMPs of both *Ureaplasma* stimulated the gene expression of IL-1β, TNF-α, TLR2 and TLR4 after 6 and 12 h following infection in macrophages. These lipoproteins appear to be important compounds to modulate the expression of the Pro-inflammatory cytokines in *U. diversum* infections. In fact, Chambaud (Chambaud et al., 1999) and You (You et al., 2006) consider LMPS to be the main virulence factors of *Mollicutes*, playing a central role in establishing an inflammatory response. *M. genitalium* LAMPs can stimulate TNF-α and IL-1β production in human monocytes (Qiu et al., 2007). This is the first report regarding the inflammatory response induced by *U. diversum* LAMPs,; therefore, further studies are needed to better understand the role of these lipoproteins in the immunopathogenesis of *U. diversum*.

Several studies with mollicutes have described the involvement of IL-1β and TNF-α both *in vivo* (Muneta et al., 2008; Wang et al., 2016) and *in vitro* (Hwang et al., 2008; Damte et al., 2011) assays. Herein, the inhibitors of TLR2, TLR4 and NF-κB induced a significant reduction of IL-β and TNF-α release from bovine macrophages when exposed to *U. diversum.* Other studies have shown the importance of NF-κB to modulate the Pro-inflammatory response in *Mollicute* infections. LAMPs derived from the same mollicute also activated NF-κB through TLR1, TLR2, and TLR6 (He et al., 2009). This also occurred in THP-1 cells infected with *M. pneumoniae* (Shimizu et al., 2005) and macrophages infected with *U. parvum* LAMPs (Shimizu et al., 2008b). The results of the present study indicate that the expression of IL-1β and TNF-α in macrophages infected with *U. diversum* and UdLAMPs did not follow this pattern of signaling, as TLR4 blockade also inhibited the expression of these cytokines. Thus, it can be suggested that *U. diversum* interacts with TLR4 to induce cytokine gene expression. The use of specific TLR2 (Pam3CysSK4) and TLR4 (LPS) ligands contributes to this conclusion. TLR4 signaling following *Mollicutes* infections is poorly described. It has been described that, following for *M. arthritidis* infection in macrophages, both TLR2 and TLR4 interact with HLA-DR and increase the binding and presentation of antigens to T cells (Shio et al., 2014). Moreover, *U. urealyticum* LAMPs have been shown to interact with TLR2 and TLR4 leading to an inflammatory response (Peltier et al., 2007).

We demonstrated a higher TLR2, TNF-α and IL-1β gene expression in bovine blastocysts experimentally infected with *U. diversum*. Cytokines are mediators and important immune regulators at the maternal-fetal interface of mammals. Cytokines secreted by the embryo are responsible for mechanisms related to its elongation and subsequent implantation (Shirasuna et al., 2012). Induction of TNF-α and IL-1β gene expression in the Co-culture of blastocysts with primary culture of bovine oviduct cells after challenge with LPS was observed. This fact suggests the existence of an early signaling system to respond to bacterial infections (Ibrahim et al., 2015). The expression of these inflammatory cytokines result in lower embryo survival and quality, and delayed embryo development (Jackson et al., 2012; Ibrahim et al., 2015). The importance of the activation of TLRs 2 and 4 during infections in cells of the bovine reproductive system was mentioned in different studies (Turner et al., 2012, 2014; Sheldon et al., 2014; Lüttgenau et al., 2016).

The present study demonstrated for the first time the presence of *U. diversum* inside hatched and intact bovine blastocysts. A characteristic of *in vitro* embryos is that they have a thinner ZP and this can make them more susceptible to infections. However, Britton et al. (1987) detected this *Ureaplasma* on the surface of the ZP with a possible transmission during embryo transfer. According to Smits et al. (1994), *U. diversum* may disturb the oviduct cells and embryo implantation and development. As observed in other *Mycoplasmas* (Baseman and Tully, 1997; Dallo and Baseman, 2000; Winner et al., 2000), Marques et al. (2010) demonstrated the invasion of *U. diversum* in Hep-2 cells and its high phospholipase C activity (Rottem, 2003). It is suggested that phospholipase C activity promotes damage to the host cell membrane and consequent bacterial invasion (Andreev et al., 1995; Rottem and Naot, 1998). The invasion of *U. diversum* in bovine blastocysts in this study clearly indicates the interference of this mollicute in bovine reproduction and highlights the risks to the offspring birth.

## Conclusion

Thus, the present study demonstrated that both *U. diversum* and UdLAMPs stimulate expression of IL-1β and TNF-α. Based on our results, *Ureaplasma* or UdLAMPs interact with TLR4. This interaction promotes activation of NF-κB, which in turn stimulates the expression of Pro-inflammatory cytokines (IL-1β and TNF-α) in macrophages. This is the first study to evaluate the immunological response generated against *U. diversum* and UdLAMPs in bovine macrophages, and also the first to show that the signaling leading to Pro-inflammatory profile expression depends on TLR4 and NF-κB. It was also found that *U. diversum* invades bovine blastocysts, without causing cytopathic effects, demonstrating that, as a consequence of this invasion, this microorganism is likely to persist in infected embryo cells, possibly preventing implantation of the embryo or causing future damage to the fetus. These results may contribute to a better understanding of the immunomodulatory activity and pathogenicity of this infectious agent in clarifying the inflammatory picture in the reproductive tract of infected cows, as well as in the better understanding of the resulting reproductive disorders.

## Ethics Statement

The study was approved by the Animal Research Committee the University of São Paulo, Brazil and by the Animal Research Committee the Federal University of Bahia.

## Author Contributions

MS-J, IR, AG, JT, and LM conceived and designed the experiments. MS-J, BB, CS, LB, EQ, EB, MT, and LDS macrophages isolation and assays. IR, AS, and MD’A blastocyst isolation. IR, MB, GC, LS, and FN blastocyst assays. IR, AM, and GM-S confocal analysis. MS-J, GC, TDS, and LM gene expression. MS-J, IR, GC, BB, JT, and LM analyzed the data. MD’A, GM-S, JT, and LM contributed reagents, materials, and analysis tools. MS, IR, AG, GC, AS, JT, and LM wrote the paper. All authors read and approved the final manuscript.

## Conflict of Interest Statement

The authors declare that the research was conducted in the absence of any commercial or financial relationships that could be construed as a potential conflict of interest. The reviewer HH and handling Editor declared their shared affiliation.
